# Respiratory syncytial virus associated hospitalizations in preterm infants of 29 to 32 weeks gestational age using a risk score tool for palivizumab prophylaxis

**DOI:** 10.1007/s10096-016-2891-6

**Published:** 2017-01-11

**Authors:** B. Resch, V. S. Bramreiter, S. Kurath-Koller, T. Freidl, B. Urlesberger

**Affiliations:** 1 Division of Neonatology, Department of Pediatrics and Adolescent Medicine, Medical University of Graz, Auenbruggerplatz 34/2, 8036 Graz, Austria; 2 Research Unit for Neonatal Infectious Diseases and Epidemiology, Medical University of Graz, Graz, Austria

## Abstract

To evaluate the efficacy of palivizumab in infants of 29 to 32 weeks of gestational age (GA) based on a risk score tool developed for Austria. Retrospective single-center cohort study including all preterm infants of 29 (+0) to 32 (+6) weeks of GA born between 2004 and 2012 at a tertiary care university hospital. Data on RSV-related hospitalizations over the first 2 years of life were analyzed and compared between those having received palivizumab and those without. The study population was comprised of 789 of 816 screened infants, of whom 262 (33%) had received palivizumab and 527 (67%) had not. Nine of 107 rehospitalizations (8.4%) in the palivizumab group compared to 32 of 156 rehospitalizations (20.5%) in the group without prophylaxis were tested RSV-positive (*p* = 0.004; OR 0.356 [CI 90% 0.184–0.689]). Proven and calculated RSV hospitalization rate was 3.1% (8/262) in the palivizumab group and 5.9% (31/527) in the group without (*p* = 0.042; OR 0.504 [CI 90% 0.259–0.981]). Increasing number of risk factors (up to three) increased the RSV hospitalization rate in infants with (6.1%) and without (9.0%) prophylaxis. RSV-associated hospitalizations did not differ between groups with regard to length of stay, severity of infection, age at hospitalization, demand of supplemental oxygen, need for mechanical ventilation, and admission rate to the ICU. A risk score tool developed for infants of 29 to 32 weeks of gestational age led to a reduction of RSV-associated hospitalizations without influencing the severity of disease.

## Introduction

Respiratory syncytial virus (RSV)-related hospitalization rates have been reported to range from 2 to 18% for preterm infants of equal or less than 32 weeks of gestational age (GA) without bronchopulmonary dysplasia (BPD), and from 7.6 to 59% for those having diagnosis of BPD [1]. Our own data from an observational multicenter nation-wide study of preterm infants of 29 to 32 weeks of GA revealed a hospitalization rate of 4.5% (36/801), with differences between the two included RSV seasons of 3.9% compared to 7.8% (*p* = 0.042) [2]. This relatively low RSV hospitalization rate on the one hand and a nation-wide heterogeneity of RSV prophylaxis reimbursement by the insurance companies in Austria on the other hand led to a revision of the initial Austrian guidelines for palivizumab prophylaxis [3] that had adopted until then the AAP guidelines [4]. Additionally, a RSV risk score tool was developed by an experts’ round to tailor palivizumab prophylaxis to the infants at highest risk for RSV-related hospitalization in this age group [5]. Risk factors included in the model were based on literature findings with regard to RSV risk factors summarized elsewhere [6, 7]. Definitively, the experts’ round [5] discussed on the basis of the risk factors found by the Austrian RSV 29-32 Study Group [2] and their own data [8].The discussion was further influenced by the findings of the PICNIC [9] and IRIS study [10] in preterm infants of 33 to 35 weeks gestational. Some unresolved issues in the discussion of different findings in both studies [11] were simplified by the committee. Thus, briefly, a birth weight below 1,500 grams, presence of neurological disease, discharge between October 1 and March 31, siblings of preschool and school age, multiple birth, day-care attendance, low socioeconomic status/crowding, and passive tobacco smoke exposure were included in the model (see Table 1). Four points or more qualified an infant for palivizumab prophylaxis. Presence of BPD, hemodynamically significant congenital heart disease, and immunodeficiency automatically were associated with 4 points, thus fulfilling the criteria for palivizumab prophylaxis [5]. The introduction of this RSV risk score tool led to homogeneous reimbursement by all Austrian insurance companies in this population.

**Table 1:** RSV risk score for immunoprophylaxis with palivizumab in preterm infants of 29 to 32 weeks of gestational age and chronological age below 6 months [5]

Risk factor	Score
Gestational age 29–32 weeks and chronological age below 6 months before onset of RSV season	2
Neurological disease*	1
Birth weight < 1500 grams	1
Discharge between October 1 and March 31	1
Siblings of preschool and school age	1
Multiple birth	0.5
Day care attendance	0.5
Low socioeconomic status/crowding	0.5
Passive tobacco smoke exposure	0.5
Bronchopulmonary dysplasia	4
Immune deficiency syndrome	4
Congenital heart disease**	4

4 points and more qualify for palivizumab prophylaxis through the first RSV season

* Including intra-/periventricular hemorrhage, periventricular leukomalacia, hydrocephalus, cerebral infarction

** According to the cardiologist’s recommendation of being hemodynamically significant

Aim of the study was to evaluate the efficacy of this RSV risk score tool in preterm infants of 29 to 32 weeks of gestational age.

## Methods

### Study population

Retrospectively, all preterm infants of 29 (+0) to 32 (+6) weeks of gestational age born between January 1, 2004 and December 31, 2012 at the Department of Pediatrics of the Medical University Graz, a tertiary care center in the southern part of Austria, were included for analysis. The study was approved by the ethic committee of the Medical University of Graz (number 27-006 ex 14/15) and started in April 2014.

Children were all followed over 2 years including at least two consecutive RSV seasons from November to April according to long-term epidemiological data from Austria [12]. Thus, for every single child included in the study, follow-up over two RSV seasons was provided. Patients were excluded for analysis if they were lost to follow-up during the first 2 years of life, or if death occurred during neonatal hospitalization or follow-up. Possible death due to RSV infection was included in the analysis.

#### Data collection

Data were collected from the local electronic database openMedocs with regard to gender, date of birth, gestational age, birth weight, small for gestational age (defined as birth weight below the 10th percentile), month of discharge, diagnosis of BPD (defined as oxygen dependency at 36 weeks postmenstrual age), neurological disease (intra-/ periventricular hemorrhage, periventricular leukomalacia), presence and number of siblings, multiple birth, tobacco smoking during pregnancy, crowding (more than three persons living under poor conditions), and prescription of palivizumab prophylaxis as documented in the medical charts according to the Austrian recommendations for RSV immune prophylaxis in preterm infants with and without BPD [5].

#### RSV risk score

With regarding to our study population, recommendations for palivizumab did not change throughout the study period. The RSV risk score for preterm infants of 29 to 32 weeks of gestational age is depicted in Table 1 [5]. A score of 4 or more points qualified an infant for palivizumab prophylaxis. The risk score was based on literature findings and experts’ rounds as described in the introduction section. The risk for preterm infants of 29 to 32 weeks gestational age to exhibit a hospitalization due to RSV disease was deemed to be at least twice as high as the risk for a term and healthy young infant (therefore 2 points). For children with severe underlying diseases, the risk was calculated to be 4 times higher (BPD, CHD, etc., all 4 points). Major risk factors were suggested to increase the risk by 1 time (1 point for neurological disease, birth weight < 1500 grams, discharge between October 1 and March 31, or siblings of preschool and school age). Crowding and low socioeconomic status were defined according to Simoes [13]. Otherwise important factors that were somehow difficult to find out during the parents interview when evaluating the risk score tool (low socioeconomic status/crowding, and passive tobacco smoke exposure) therefore received only 0.5 points as did the factors of day care attendance (well known, but in Austria only marginally present — 1.3% in the Austrian RSV 29-32 study [2]) and multiple birth with few studies on its evidence [14]). It was calculated that nearly all infants of 29 to 32 weeks of gestational age discharged during the RSV season would have received palivizumab. In total, it was suggested that not more than 50% of the cohort would be eligible for palivizumab, giving reassurance that the insurance companies would accept remuneration of this model. Acceptance by the insurance companies was at least the main incitement for the development of the risk score tool.

#### RSV hospitalization

RSV hospitalization was defined as hospitalization associated with LRTI and a positive RSV test result. RSV testing was performed from nasopharyngeal aspirates using RSV-ELISA (Directigen EZ RSV Test, Becton Dickinson, USA; sensitivity 66.7–87.2%; specificity 85.5–91.6%). Criteria for hospitalization in case of LRTI in general included young age (less than 6 months), signs and symptoms of respiratory distress, history of prematurity or underlying disease, and/or feeding difficulties. The calculated RSV hospitalization rate included infants with diagnosis of bronchiolitis (LRI score ≥ 3) not tested for RSV during the typical RSV season, of whom 70% were assumed to be RSV-positive. This rate was based on the evidence from the literature that RSV accounts for 50 to 80% of all hospitalizations for bronchiolitis during seasonal epidemics [15,] and according to its use in comparable studies [16].

Data were collected regarding days of hospitalization due to respiratory illness, age at admission in months, month of RSV hospitalization, days of oxygen requirement, days at the intensive care unit (ICU), days of respiratory support (either nasal continuous positive airway pressure, or mechanical ventilation). In cases of palivizumab prophylaxis, we did not evaluate the number of doses of each recipient for further analysis. Severity of LRTI was measured using the modified lower respiratory illness/infection (LRI) score ranging from 1 to 5 [17]. In detail: 1 = upper respiratory tract infection, 2 = mild LRTI without respiratory distress (tachypnea, retractions), 3 = moderate LRTI with respiratory distress without oxygen demand, 4 = severe LRTI with oxygen demand, and 5 = assisted ventilation.

#### Statistical analysis

Statistical analysis was performed using Excel (Microsoft Office, Excel 2013) and SPSS (IBM SPSS Statistics 22). For categorical data, chi-square test or Fisher’s exact test, and for numerical data *t*-test or Mann–Whitney U test were used as appropriate. Normality assumption was checked using the Shapiro–Wilk test. Odds ratio and 90% confidence intervals were calculated using the software package CIA (Confidence Interval Analysis; version 2.0.0: Statistics with confidence; London: BMJ Publishing Group, 2000). Statistical significance was set at *p* < 0.05.

## Results

### Study group

**Fig. 1:**
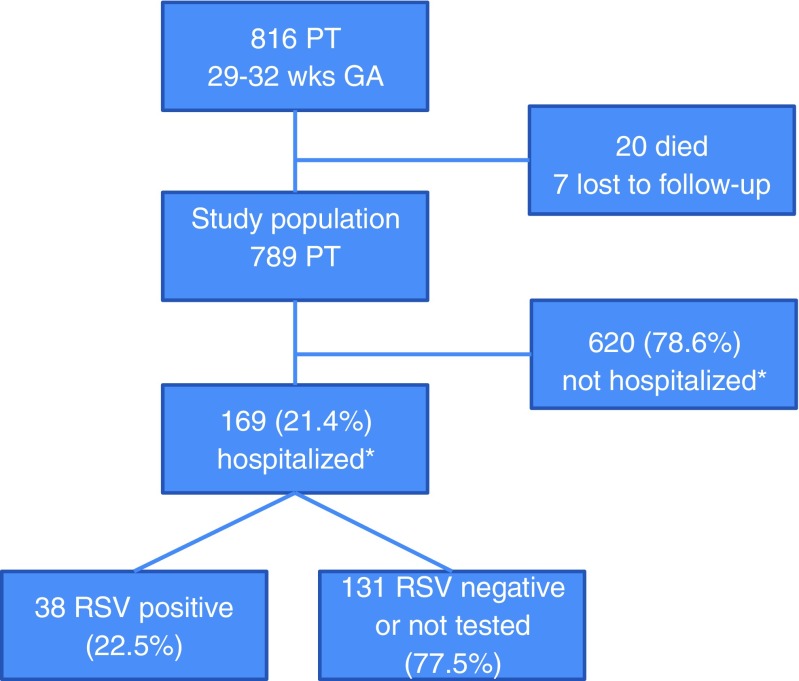
Flowchart of screened and included preterm infants of 29 to 32 weeks of gestational age born 2004 to 2012, and documentation of hospitalizations due to respiratory illness within first 2 years of life

During the study period, 816 preterm infants of 29 to 32 weeks of GA were born. Twenty infants died, seven were lost to follow-up, and thus the study population comprised 789 infants. One-hundred and sixty-nine infants (21.4%) were hospitalized due to respiratory illness during the first 2 years of life for a total of 263 times. Thirty-eight infants were tested RSV-positive (see Fig. 1), representing a total RSV hospitalization rate of 4.8%.

### Rehospitalizations

Palivizumab prophylaxis was given to 262 infants (33%), of whom 70 (26.7%) were hospitalized (107 times) due to respiratory illness. Of 527 infants without palivizumab prophylaxis, 99 (18.8%) were hospitalized (156 times) due to respiratory illness (*p * = 0.005). Nine of 107 rehospitalizations (8.4%) in the palivizumab group, compared to 32 of 156 rehospitalizations (20.5%) in the group without prophylaxis, were tested RSV-positive (*p * = 0.004; OR 0.356 [CI 90% 0.184–0.689]).

### Effect of palivizumab prophylaxis

In the palivizumab group, seven infants (2.7%) were hospitalized due to proven RSV infection during the first RSV season, two outside the first RSV season, and zero during the second RSV season. In the group without prophylaxis, 25 infants (4.7%) were hospitalized due to proven RSV infection during the first RSV season (one infant exhibited two RSV infections), six outside the first RSV season, and zero during the second RSV season. Two infants in the palivizumab group and seven in the group without prophylaxis had no RSV test done (5.3%); thus, one and five cases respectively were calculated to be RSV-positive. Proven and calculated RSV hospitalization rate during the first RSV season was 3.1% (8/262) in the palivizumab group and 5.9% (31/527) in the group without (*p* = 0.042; OR 0.504 [CI 90% 0.259–0.981]). This is a 48% relative risk reduction.

### Seasonal distribution

Forty-one hospitalizations due to proven RSV infection occurred in 38 infants during the study period. RSV hospitalizations peaked in January (nine; 22%) followed by March (seven; 24%), and February and December (each six; 15%). Ten percent of RSV hospitalizations (*n* = 4) occurred before onset of the first RSV season, 80% (*n* = 43) during the first, and 10% (*n* = 4) between first and second season, none during the second season.

### Risk factors

Palivizumab led to a significant reduction of proven RSV LRTI in boys (*p*= 0.037), multiples ((*p* = 0.026) and infants discharged during the RSV season(*p* = 0.044). The risk of RSV hospitalization increased with the number of risk factors: presence of one risk factor was associated with a 1.4% RSV hospitalization rate, two risk factors with a 2.6% rate, three with a 9% rate, and four with an 8.7% rate in the group without prophylaxis and 0%, 4.6%, 6.1%, and 1.9%, respectively, in the palivizumab group.

### Hospitalization data

There were no differences with regard to length of stay, demand of oxygen, need of mechanical ventilation, admission rate to the ICU, and severity of infection measured by LRI scores between groups; details are provided in Table 2.

**Table 2:** Data on RSV hospitalization comparing seven infants (29 to 32 weeks of gestational age) with and 26 without palivizumab prophylaxis during the first RSV season (November till April)

RSV hospitalization	Palivizumab group (*n* = 7)	No prophylaxis (*n* = 26)	*P*-value
Length of stay (days)	8 (4–15)	8 (1–21)	0.62
Days on supplemental oxygen	0 (0–12)	0 (0–16)	0.50
Infants with supplemental oxygen	1 (14)	9 (35)	0.30
Mechanical ventilation (days)	0 (0–10)	0 (0–16)	0.91
Infants with mechanical ventilation	1 (14)	5 (19)	0.76
Stay at the ICU (days)	0 (0–15)	0 (0–21)	0.81
Admission to the ICU	2 (29)	6 (23)	0.76
Chronological age (months)	2 (1–6)	4 (1–11)	0.23
LRI score	3 (2–5)	3 (1–5)	0.95

Data are given as median (range) or *n* (%)

ICU = intensive care unit; LRI score = lower respiratory tract infection score (1–5)

## Discussion

Our study demonstrated efficacy of palivizumab prophylaxis in a population of preterm infants of 29 to 32 weeks’ gestational age using a RSV risk score tool developed for Austria [5]. It was the first evaluation of this risk score tool. The severity of RSV disease mainly measured by hospitalization data was not influenced by palivizumab prophylaxis. Additionally, increasing number of risk factors (up to three risk factors) increased the risk for RSV hospitalization irrespective of palivizumab prophylaxis.

The Impact study group reported on a RSV hospitalization rate in the palivizumab group of 4.8% [18]. This was the rate we observed in the non-prophylaxed infants. Thus, our further reduction of the rate of proven RSV hospitalizations by 2% is still remarkable. In a real-world perspective, Homaira et al [19] reported on reduction rates of RSV hospitalizations ranging from 1.2% to 12.4%. Follow-up of 13,310 infants from the Canadian CARESS study [20] revealed a RSV rehospitalization rate of 1.47%. Romero et al. [21] reported on rates lying between 1.5% and 2.9% over a four-season follow-up from the US. Pedraz et al. [22] found a rate of 3.95% in Spain, and in Germany the rate was 2.5% from a cohort of 10,686 infants collected from a palivizumab registry [23]. Preterm infants born 29 to 32 weeks of GA without BPD who received palivizumab prophylaxis had a low 1.8% incidence of RSV-related hospitalizations in a European multicenter study enrolling infants younger than 6 months of chronological age over the 2000/2001 RSV season [24] — a finding comparable to that observed in the pivotal trial conducted in North America and the UK [18]. The RSV rehospitalization rate in our non-prophylaxed group was astonishingly low compared to other studies reporting on rates ranging from 5.0% to 13.3% [18, 20, 25, 26].

Increasing numbers of risk factors increased the rates of RSV hospitalizations in our study, as confirmed by others [25, 27]. This finding affirms the effectiveness of several risk score tools in different populations [28–32]. A recent meta-analysis [33] found eight among 18 risk factors being significantly associated with RSV associated acute LRTI including prematurity (odds ratio 1.96), low birth weight (1.91), male gender (1.23), siblings (1.60), maternal smoking (1.36), a history of atopy (1.47), no breastfeeding (2.24), and crowding (1.94). Illness severity was not predicted accurately in a cohort of term infants [34]. In contrast to other reports [19], we were unable to document an influence of palivizumab on length of stay, demand of oxygen, need of mechanical ventilation, admission rate to the ICU, or severity of infection measured by LRI scores between groups.

Limitations of the study include the retrospective study design and the single-center analysis enrolling infants over a large period of time. On the other hand, the single-center experience presented here includes homogeneous follow-up data due to the geographical catchment area. The strength of this single-center study is the high rate of RSV testing, demonstrated by the small number of cases that had to be included in the calculated RSV hospitalization rate. Even in prospective studies, not all infants were tested for RSV [16]. The model used in our study for the calculated RSV hospitalization rate is well established in the literature [16].

Interestingly, the second season revealed no case of RSV hospitalization, supporting the recommendations for palivizumab prophylaxis in this population focusing on infants younger than 6 months of chronological age. In total contrast to existing guidelines on palivizumab prophylaxis recently, the AAP reported that available data for infants born at 29 weeks, 0 days gestation, or later did not identify a clear gestational age cutoff for which the benefits of prophylaxis might be clear, and for this reason, infants born at 29 weeks, 0 days gestation, or later are not universally recommended to receive palivizumab prophylaxis [35]. This is an astonishing revision of the former recommendations by the AAP that is not based on epidemiologic data and the proven burden of RSV disease in this particular gestational age group [1].

Approximately one quarter of RSV hospitalizations occurred outside the typical RSV season and, hence, will not be influenced by palivizumab prophylaxis. This is a well-known phenomenon, as is the year-to-year variability of RSV activity [12, 25]. Thus, epidemiological studies are requested to cover at least two or more RSV seasons.

In conclusion, the Austrian risk score tool — developed for palivizumab prophylaxis in infants of 29 to 32 weeks of GA — led to a reduction of RSV associated hospitalizations in our single-center retrospective analysis without influencing the severity of RSV disease.
